# The Human Release Hypothesis for biological invasions: human activity as a determinant of the abundance of invasive plant species

**DOI:** 10.12688/f1000research.3740.2

**Published:** 2014-12-12

**Authors:** Heike Zimmermann, Patric Brandt, Joern Fischer, Erik Welk, Henrik von Wehrden

**Affiliations:** 1 Institute of Ecology, Faculty of Sustainability, Leuphana University, Lüneburg, 21335, Germany; 2 Centre for Methods, Leuphana University, Lüneburg, 21335, Germany; 3 Institute of Biology, Geobotany and Botanical Garden, Martin-Luther-University Halle Wittenberg, Halle, 06108, Germany; 4 Research Institute of Wildlife Ecology, Vienna, 1160, Austria

## Abstract

Research on biological invasions has increased rapidly over the past 30 years, generating numerous explanations of how species become invasive. While the mechanisms of invasive species establishment are well studied, the mechanisms driving abundance patterns (i.e. patterns of population density and population size) remain poorly understood. It is assumed that invasive species typically have higher abundances in their new environments than in their native ranges, and patterns of invasive species abundance differ between invaded regions. To explain differences in invasive species abundance, we propose the Human Release Hypothesis. In parallel to the established Enemy Release Hypothesis, this hypothesis states that the differences in abundance of invasive species are found between regions because population expansion is reduced in some regions through continuous land management and associated cutting of the invasive species. The Human Release Hypothesis does not negate other important drivers of species invasions, but rather should be considered as a potentially important complementary mechanism. We illustrate the hypothesis via a case study on an invasive rose species, and hypothesize which locations globally may be most likely to support high abundances of invasive species. We propose that more extensive empirical work on the Human Release Hypothesis could be useful to test its general applicability.

## Introduction

Biological invasions can threaten ecosystems ^1^, economies ^2^, and human health ^3^. The Scientific Committee on Problems of the Environment (SCOPE) put biological invasions on top of its research agenda in 1983 ^4^. Since then, the field of invasion ecology has rapidly gained momentum. The number of publications dealing with biological invasions has increased a hundredfold in less than two decades ^5^. Several journals are partly (e.g. *Diversity and Distributions, Natural Areas Journal*) or fully (e.g. *Biological Invasions, Invasive Plant Science and Management, NeoBiota*) devoted to research, management and policy issues related to invasive species. However, despite a growing body of knowledge on biological invasions, difficulties remain in predicting invasion success ^6^.

Within Europe, the distribution of people is strongly related to the number of alien species. Presumably, this reflects that biological invasions are aided by human transport and that species establishment is facilitated by human disturbance ^7^. Nevertheless, at the global scale, the proportion of widely distributed alien plant species (relative to all species) is far lower in Europe than in North America – despite Europe’s long history of trade and therefore a longer residence time of alien plants ^8^. The observation that Europe serves as a global contributor of alien plant species, whereas North America seems to be a better recipient, has sparked the concept of biological resistance, which explains invasion success or failure in relation to the traits of the native flora ^9^. An additional important consideration, which has not been assessed to date, could be that Europe also has a higher proportion of landscapes that are actively managed by humans than, for example, the Americas, Australia and Africa ^10^. To date, extensive data on the abundance of invasive alien species is widely lacking. Existing approaches to predict invasion patterns in response to anthropogenic global change have focused primarily on the development of novel ecosystems ^11^, and alien species richness ^12^. Based on this, it is now widely acknowledged that systems containing high numbers of alien species tend to be those created and sustained by humans.

In this paper, we do not focus on species richness. Rather, we propose that the *abundance* of an alien species in a given landscape can be (at least partly) explained by the level of active landscape maintenance by humans – that is, the active, continuous and on-going management by people. We term this hypothesis the Human Release Hypothesis. As discussed in detail below, the Human Release Hypothesis states that the abundance of invasive species may be partly explained by the level of human activity or landscape maintenance, with intermediate levels of human activity providing optimal conditions for high abundance. We define intermediate levels of human activity as activity patterns defined by sporadic disturbance events that are followed by long periods lacking active management, such as fallowing or abandonment. In contrast, regions with high levels of human activity frequently experience active management, such as weeding, hedge trimming or mowing of field margins.

Unlike the Disturbance Hypothesis and the Intermediate Disturbance Hypothesis, which explain patterns of establishment of invasive species ^13^ and patterns of native species diversity in relation to land use ^14^, the Human Release Hypothesis specifically addresses the effect of land use on the abundance of alien species that are already established in particular areas outside their native ranges. Furthermore, in areas where Human Release takes place, single disturbance events may occur, but alien species can grow large populations because of a lack of active and continuous landscape maintenance. Finally, we propose that the Human Release Hypothesis can also explain why some species that are highly abundant in their invasive range have relatively low abundance in their native range. Such differences in abundance between native and invasive ranges could at least partly be explained by different patterns in land use in the two sets of ranges.

We first discuss how the Human Release Hypothesis fits into the context of other key hypotheses in invasion ecology. We then illustrate the hypothesis via a case study on a global invader, the sweetbriar rose ( *Rosa rubiginosa* L.). Finally, we assess how the Human Release Hypothesis may be integrated into biological invasion research, and we hypothesize which locations worldwide may be particularly prone to supporting high abundances of invasive species.

## The Human Release Hypothesis in the context of other invasion hypotheses

According to Richardson *et al.* (2000) ^15^, an invasive terrestrial plant species is a naturalized alien species that produces reproductive offspring, often in very large numbers, at considerable distance from parent plants, and thus has the potential to spread over extensive areas. A key question in invasion ecology is how the interaction of species traits with environmental characteristics predicts invasion success, including both establishment and abundance in the new environment ^6^. We focus our hypothesis on the latter issue, that is, the abundance of an alien species resulting in dominating populations in the new range ^16^.

**Figure 1.**
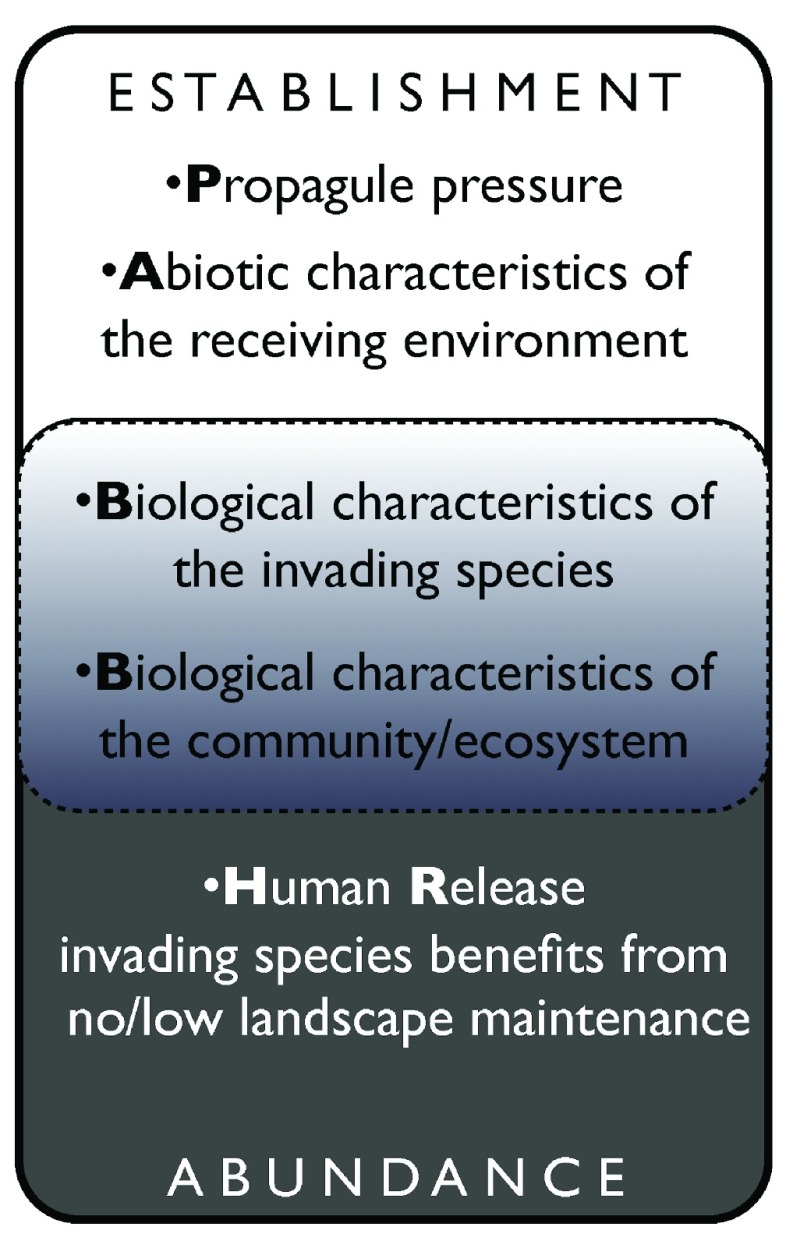
Incorporating our hypothesis into the PAB framework. The establishment and abundance of invasive plant species are explained by different mechanisms, which have been summarized by Catford *et al.* (2009) ^16^ in the PAB framework (see text for details). However, the biological characteristics of a given invading species and of its new environment only partly explain the abundance of established invasive populations. We argue that additional insights can be gained via the Human Release Hypotheses, which can complement the existing PAB framework.

Catford *et al.* (2009) ^17^ summarized 29 leading hypotheses predicting invasion success and integrated them into the PAB-framework ( Figure 1). This framework considers the size and frequency of introductions (i.e. propagule pressure, P), ecosystem invasibility based on abiotic characteristics of the new environment (A), and biotic characteristics of an invasive species and its recipient community (B). By testing the validity and importance of each factor, the main driver of a successful invasion can be identified. The Human Release Hypothesis applies after a successful invasion has already been accomplished, because it focuses on the abundance of successful invaders.

So far, human influence has been recognized as a mediating influence on the process of invasion, but not as a key of the abundance of invasive species. Human influence thus has been considered primarily during the establishment stage. For example, human action can increase propagule pressure ^18^ and multiple introduction events make establishment more likely, because species have a higher chance to encounter suitable environmental conditions ^19^. At this stage of the invasion process large-scale planting of alien species could also contribute to the abundance of invasive species, as demonstrated for tree species ^20^. Multiple introductions of the same species also can lead to higher genetic diversity ^21^. However, examples exist of successful invaders with low genetic diversity ^22^, and stemming from single or few introduction events, suggesting that propagule pressure is only one of many variables explaining invasion patterns ^23^.

With respect to abiotic conditions, invasion is facilitated if species are pre-adapted to their new environment, for example due to a similar climate in the new environment ^24^. Like propagule pressure, pre-adaption is not a necessary precondition for successful invasion, because climatic niche shifts have been reported for invasive species ^25^. Disturbance events also provide windows of opportunity for invasive species ^25^. Many invasive plant species are adapted to exploit temporarily favourable conditions through their short life cycles, rapid growth, high reproductive allocation, persistent soil seed banks and rapid germination (the Ideal Weed Hypothesis) ^27^. All these traits are also of advantage in systems where frequent weeding or mowing is practiced. Therefore, species pursuing this competitive ruderal strategy could profit twofold from Human Release.

Finally, biotic characteristics of the recipient community may involve the absence of natural enemies. The Enemy Release Hypothesis explains invasion success as a function of alien species having escaped their natural enemies, allowing them to allocate resources to growth and reproduction rather than defence ^28^. This would make alien plants stronger competitors. In the context of the Intermediate Disturbance Hypothesis, which proposes higher species diversity at intermediate frequencies or intensities of disturbance (see Wilkinson, 1999) ^14^, alien plants are likely to have the greatest impact on community diversity when resources become limited and plant diversity is highest, by co-opting more resources ^29^.

In parallel to the Enemy Release Hypothesis, here, we propose the Human Release Hypothesis. It describes a situation where alien species have escaped relatively higher levels of human landscape maintenance that is characteristic within their native ranges. Changing patterns of land use are widely recognized to increase opportunities for introduced species to establish and spread ^30^, but already prevailing patterns of land use intensity also should be expected to influence the populations of species – both in their native and introduced ranges. This is because highly intensive land use by humans (such as in many parts of Western Europe) often corresponds to high levels of active landscape maintenance – which translates into little available habitat for both native and introduced species, as well as high levels of active weed control. At the other end of the spectrum of human land use intensity, we hypothesize that pristine natural habitats also offer few windows of opportunity for alien species to establish (the Biotic Resistance Hypothesis) ^31^. Thus, we hypothesize that the abundance of invasive species should be highest in between these two extremes – namely in extensively used landscapes characterized by frequent fallowing, low levels of weed control, high heterogeneity, and many disturbed edges of small farmland patches ^32^. Such landscapes are where “human release” should contribute to optimal conditions for invasive species to establish large populations.

While existing hypotheses explain the establishment and naturalization process of invasions, little work has attempted to explain the (potential) abundance of invasive species in their new environments. Part of this gap may be effectively addressed by the Human Release Hypothesis ( Figure 1).

## Case study on an invasive rose

To illustrate the plausibility of the Human Release Hypothesis, we present findings at two scales on the invasion success of *Rosa rubiginosa*, a shrub native to Eurasia and invasive in Australia, New Zealand, South Africa, North and South America (see Dataset 1 and Supplementary Figure S1). We show that existing hypotheses could not fully explain the invasion patterns observed for this species, and we argue that the Human Release Hypothesis could help to fill this explanation gap. First, we synthesize previous cross-continental case studies that compared plant performance between invasive populations in Central and Southern Argentina with native populations in Spain and Germany (for more details see Zimmermann *et al.*, 2012) ^33^. Second, we compare climatic conditions as well as land use and human population density between invasive and native *R. rubiginosa* populations at a global scale. In combination, our findings suggest the Human Release Hypothesis may be a useful complementary hypothesis to other existing hypotheses in invasion biology ( Table 1).

**Table 1.** Incremental approach to identify the most influential mechanisms for the invasion success of *Rosa rubiginosa* in Argentina. ( ^a^Cavallero & Raffaele 2010, ^b^Zimmermann *et al.* 2010, ^c^2011, ^d^2012, ^e^Hirsch *et al.* 2011, ^f^present publication).

Hypothesis	Mechanism	Case study
Propagule Pressure	Multiple introductions into new range make establishment more likely and secure high genetic diversity or large-scale planting of one particular genotype secure colonization byseed- swamping	Genetic diversity in invasive populations very low, and no records of plantations, small number of introduction events ^b, e^	REJECTED
Favorable environmental conditions	Species benefits from climatic or edaphic conditions, or vegetation characteristics in new range	Structure of vegetation matrix did not differ between ranges, edaphic conditions not favourable in invasive populations and climatic conditions vary greatly within the introduced range ^d, f^	
Enemy Release	Invasive species allocates resources no longer needed for defence to growth and reproduction	Damaged or infested leaf area high in invasive and native range and no difference in plant performance in common garden experiments ^d^	
Evolution of Increased Competitive Ability	Selection favours genotypes which have allocated freed resources, to adapting and enhancing competitive ability	Individuals from both ranges same growth rates in common garden experiments ^d^	
Ideal Weed	Invasive species share traits that facilitate invasions under particular environmental conditions	Ideal weed traits of study species: high phenotypic plasticity, clonal growth, asexual reproduction ^b, d^, that enable growth and colonization under wide range of environmental conditions	CONFIRMED
Disturbance	Disturbance events open window of opportunity for invasive species	Species occurs in invasive range across habitat types after anthropogenic or natural disturbance ^a, c^	
Human Release	Invasive species benefits from low levels of landscape maintenance	Trimming or removal of individuals only in native range, individuals in invasive range older, in invasive range lower number of people/km ^2^ as well as less residential areas and less cropland area than in native range ^d, f^	PROPOSED

**Figure 2.**
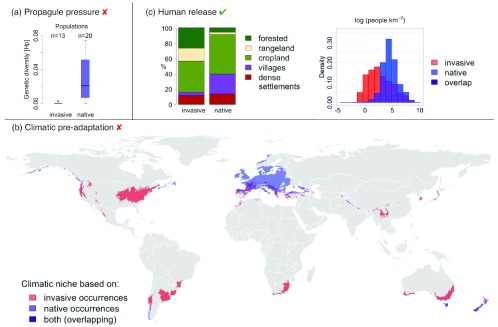
*Rosa rubiginosa* benefits from human release. ( **a**) Genetic diversity in *Rosa rubiginosa* is higher in its native Spanish and German populations than in the introduced populations in Argentina, suggesting the species did not benefit from multiple introductions (for details see Zimmermann *et al.* 2010) ^34^. ( **b**) The species does not benefit from a climatic pre-adaptation to the new range. The world map shows the species' climatic niche based on the species’ native distribution (blue) and the invasive distribution (pink). Overlap of climatic niches (purple) is minimal. ( **c**) *Rosa rubiginosa* appears to benefit from “human release” in its new range. The barplot shows the global proportions of different anthropogenic biomes ^10^ according to the location of invasive and native sweetbriar rose populations. The native range has a larger proportion of residential areas and a higher human population density (log people/km ^2^). Only 0.56% of the invasive range is wildlands, and only 0.03% of the native range.

**Figure 3.**
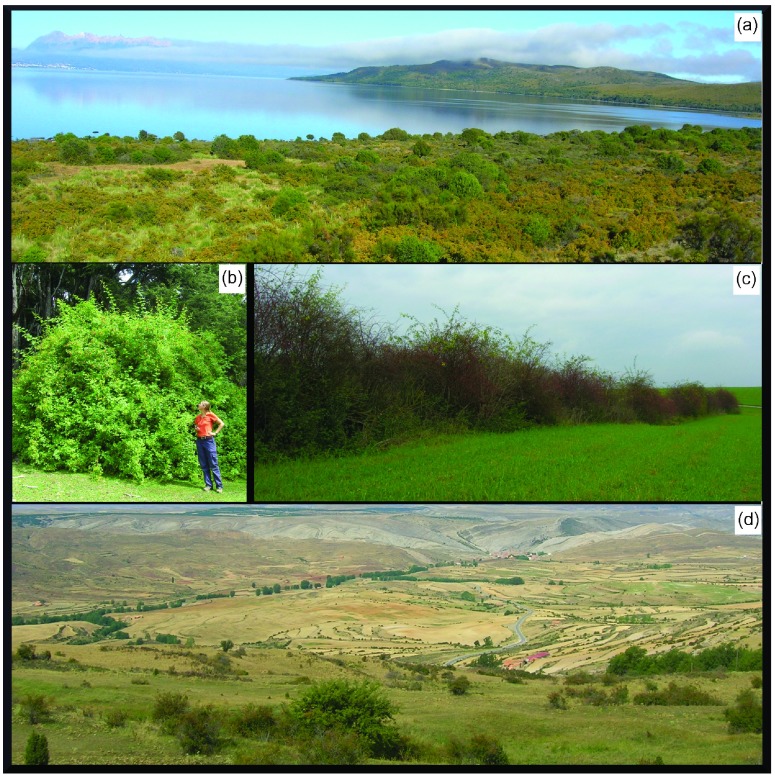
Invasive *Rosa rubiginosa* populations in Argentina (a, b) and native populations in Germany **(c)** and Spain (d). In parts of Argentina, single disturbance events have offered windows of opportunity for the species to establish populations, some of which have remained undisturbed for 30 years or longer ( **a**) ^33, 39^. The low level of human landscape maintenance means that populations can expand over vast areas and consist of hundreds of individuals ( **a**, here along the whole visible lakeside in Patagonia). ( **a**) For our study area in Patagonia we predicted that 36% of the area (5000 km ^2^) was threatened by *R. rubiginosa* invasion, across a precipitation gradient from 1400 mm/annum (mountains in the far background) to 600 mm/a ^39^. In Argentina *R. rubiginosa* shrubs have time to grow to their full size ( **b**), by contrast, many native landscapes are regularly maintained; shrubs are regularly trimmed and mostly grow in hedgerows ( **c**, Germany). Furthermore, in Germany and Spain, fewer habitats are available in landscapes dominated by agriculture and urban areas ( **d**, Spain).

*Rosa rubiginosa* has successfully invaded a range of ecosystems within Argentina (e.g. high montane grasslands, Patagonian steppe, pastures, road margins, floodplains), covering a major climatic gradient, but exhibiting low levels of genetic diversity ^34, 35^ ( Figure 2a). Low genetic diversity suggests that multiple introduction events constituting particularly high propagule pressure cannot explain the species' invasion success. Despite lower genetic diversity, populations of *R. rubiginosa* are considerably smaller in Spain and Germany than in Argentina ( Figure 3) – native populations consist of 5 to 20 individuals whereas invasive populations consist of hundreds of individuals ^33^. In addition to propagule pressure, abiotic and biotic variables also cannot fully explain the invasion success of *R. rubiginosa*. In Argentina, the species neither benefits from favourable soil conditions nor from reduced biotic resistance ^33^.

Moreover, a global climatic analysis shows that *R. rubiginosa* also does not depend or benefit from pre-adaptation to the climate of its new environment ( Figure 2b). We developed two climatic envelope models based on BioClim parameters and the occurrence of native and invasive populations respectively using the maximum entropy method ^36^ (MAXENT, see Appendix 1 and 2 in the Supplementary material). We detected a significant differentiation of realized niches between invasive and native populations based on the MAXENT model (Schoener’s D=0.31, p<0.0001; Figure 2b) as well as through a direct ordination approach ^37^ (Appendix S1, Supplementary Figure S4 in Supplementary material). Furthermore, back-projection of the climatic niche based on invasive populations points to a southern European origin. However, genetic analyses tracked the native origin of invasive Argentinean, Chilean, Australian and New Zealand populations to Central Europe ^34, 35^. Key climatic predictors therefore do not point to a climatic advantage in the invasive range, because the native genotypes were not from the climate they invaded in the alien range, but instead indicate that *R. rubiginosa* is able to thrive under a wide range of conditions (Supplementary Figure S2 and Supplementary Figure S3).

The Ideal Weed and Disturbance Hypotheses ( Table 1) partly explain the invasion success of *R. rubiginosa* in Argentina ^33, 38, 39^. However, the Enemy Release Hypothesis failed to explain abundance patterns – natural enemies appeared equally harmful to the species in the native and introduced ranges ^33^ ( Table 1). By contrast, in the invasive range, anthropogenic disturbances such as logging and burning create windows of opportunities for the rose to establish, but just as importantly, disturbance events are then followed by decades of abandonment that enable the species to become abundant.

Having considered a wide range of existing hypotheses ( Table 1), we found that additional insights into the invasion patterns of *R. rubiginosa* may be gained by the Human Release Hypothesis. This is because a key difference between native and introduced environments appears to be the level of active landscape maintenance. In the case study, we observed frequent trimming or removal of individuals only in Spain and Germany and not in Argentina, and individuals and populations in Argentina were significantly older than their native counterparts ^33, 39^. At the global scale, our analysis revealed a similar pattern (albeit at a coarser resolution; 2.5 × 2.5 arc min, Figure 2c). Native *R. rubiginosa* populations occur in areas with higher proportions of cropland, residential areas and human population densities than invasive populations ( Figure 2c). These conditions very likely correspond to a high degree of landscape maintenance, and hence little available habitat for *R. rubiginosa* in its native range. Our results at this coarse scale could also provide some explanation why, more generally, Eurasian species show less niche unfilling and more expansion in North America and Australia than do North American species in Eurasia ^40^. In addition to human mediated propagule pressure from Eurasia to North America and Australia, and a longer history of weed selection in human-disturbed landscapes in Eurasia ^40^, the higher degree of landscape maintenance in Eurasia may substantially decrease invasibility on this continent.

Dataset 1. Rosa rubiginosa L. occurrence data (occurrences_R.rubiginosa.csv, 416 kb) Presence points of R. rubiginosa in its native (N=12132) and invasive (N=1425) range derived from the literature and field data. Geographical positions are given in decimal degrees (longitude, latitude, WGS84). For the final MAXENT model we included a randomly reduced native dataset (3033 presence points) until data points were evenly distributed and no spatial autocorrelation was detected in the model residuals.

## Integrating the Human Release Hypothesis with other explanations

A key premise of this paper is that existing hypotheses that predict invasion success can be effectively complemented by the Human Release Hypothesis ( Figure 1). Our own data, of course, focused only on one species – which is enough to pose a hypothesis, but far too little to test its general usefulness. We want to emphasize that our hypothesis is complementary and acknowledge the fact that multiple interacting mechanisms often contribute to invasions ^41^. To this end, we endorse integrated testing of hypotheses, to identify if Human Release is the main driver of high abundance of invasive species. While Human Release could also be manipulated via experiments, we recommend to investigate on-site land use patterns via direct field studies, or at a global scale, drawing on appropriate land use proxies (e.g. anthropogenic biomes, human appropriation of net primary production, population census data). As demonstrated with this case study and recommended by Catford *et al.* (2009) ^17^, integrated hypothesis testing could follow the PAB framework in an incremental approach. To that effect, a top down approach, starting with the most complex scenario (PAB + HR) and then gradually eliminating non-plausible explanations, could serve to identify under what circumstances Human Release is an important driver. Furthermore, to draw universal conclusions, case studies on a single study species should be designed with multi-site sampling, as well as case studies in certain environments be conducted on multiple species ^6^. Ideally, the hypothesis should be tested by comparing the same species in its native and invasive range on ecological similar abandoned and maintained sites. However, such comparisons could be difficult since we state that land abandonment is rare in the native range of a species. If comparable sites are not available, these differences in land use between ranges can also provide insights on the validity of the Human Release Hypothesis. Studies should then focus on long-term monitoring of populations in both ranges to quantify if they are being diminished by land use practice.

On this basis, we see two research priorities that should be addressed to further scrutinize the Human Release Hypothesis so that, if appropriate, it can be integrated into invasive species management. First, additional species should be studied in both their native ranges and in different parts of their introduced ranges. Such comparisons would be useful to test the drivers of invasive species abundance and to validate (or refute) invasion patterns derived from modelling approaches ^11, 12^. We generated our hypothesis based on findings in Europe, however many invasive plant species on the American continent originate from Asia ^42, 43^, thus it would be interesting to test our hypothesis based on land use patterns from these regions. An important first clue that the Human Release Hypothesis may be relevant could be whether invasive individuals of a given perennial species are significantly older than individuals within the native range. Second, it may be useful to further investigate the relationship between landscape maintenance and human land use intensity, how it manifests in different regions, and if generalizations are possible at the global scale. The frequency and timing of weeding and trimming, as well as the prevalence of fallowing, are just two of many potential indicators for the level of active landscape maintenance.

If human release is identified as one of the most influential mechanism for invasive species abundance, this information could be transferred to management as leverage to prevent, eradicate, contain or mitigate biological invasions ^44^. Biological invasions could be prevented by implementing policies that prevent land abandonment, or promote restoration and monitoring of fallows. This demands interdisciplinary system knowledge, which can only be achieved by integrating the social and natural sciences ^45^. Especially in regions with low human population density (e.g. < 200 people km ^-2^) ^10^, land may be perceived as hyper abundant, providing ideal conditions for single disturbances followed by years without active management. If the Human Release Hypothesis gains support, this would suggest that sporadic disturbances through road construction of forest clearing may call for on-going human management in order to prevent invasive populations becoming so abundant that eradication efforts are futile.

Restoration to the original state is only possible if land use practice did not result in the crossing of an abiotic threshold with altered abiotic conditions in a way that they no longer support historic native plant communities or in the crossing of a biotic threshold, that is native species have gone extinct ^46^. In this case directional change towards novel plant communities should be considered that provide important ecosystem services and maintain conditions favourable to native communities ^47^. Invasive species could be contained by frequent weeding and trimming practice, as has been demonstrated for invasive creepers in the Seychelles ^48^ and for the native populations in our case study. Mitigation could be achieved if highly invasible areas, like fallows and set-aside land, are limited to small isolated fragments, thereby inhibiting vast invasive monocultures.

Evidently, the Human Release Hypothesis is still in its infancy, and it would be unwise to make bold management recommendations on its basis. Based on our analysis to date, preliminary insights that are relevant to managing invasive species are: (i) sparsely populated areas may face a higher risk of biological invasions than more densely populated areas; (ii) extensively managed rangelands may be more susceptible to high abundances of invasive species than intensively managed croplands; and (iii) high abundances of invasive species at landscape and regional scales could be facilitated by long periods of fallowing or land abandonment ^46^.

## Data availability

*figshare*: Dataset 1. *Rosa rubiginosa* L. occurrence data (occurrences_R.rubiginosa.csv, 416 kb). Doi: 10.6084/m9.figshare.1002067 ^49^

## Supplementary material

Supplementary Material (Version 2) The Human Release Hypothesis for biological invasions: human activity as adeterminant of the abundance of invasive plant species. Supplementary figures S1–4 and Appendices 1 and 2
Click here to access the data.
