# Supplementary material for: The Human Release Hypothesis for biological invasions: human activity as a determinant of the abundance of invasive plant species
Source: F1000Res. 2014 Dec 12;3:109. Originally published 2014 May 14. [Version 2] doi: 10.12688/f1000research.3740.2 (PMC4207246; doi:10.12688/f1000research.3740.2)
Supplement: Peer review history

# Peer review: Referee response for version 1

**DOI:** 10.5256/f1000research.4008.r6008

**Published:** 2014-09-02

**Authors:** Melisa Giorgis

**Affiliations:** 1 Instituto Multidisciplinario de Biología Vegetal, CONICET, Córdoba, Argentina

This manuscript presents a simple and interesting hypothesis about how human activities could drive the increase in abundance of invasive species. It is based on two important observations; 1. Invasive species have in general higher abundances in their new environment than in the native ranges, and 2. The patterns of invasive species differ between invaded regions. The authors realized that human land management activities explain the difference in abundance of species, between both the invader and native range and between different invader regions. Specifically they put a new role for “human activities” into the second stage of plant invasion ( Diez & Edwards, 2006). I think that it is an interesting hypothesis, which provides a new vision and background for future research on invasion ecology and conservation management. Finally, I have some suggestions that may help to a better understanding of this article and future development.

 

1. The need for a clear definition of “active landscape maintenance by human”. The authors at the end mention the frequency of weeding and trimming, as potential indicators of active landscape maintenance. But could human maintenance be defined as any human activity developed in order to sustain the same physiognomy, structure, floristic composition or/and biomass? “Active landscape maintenance” is for me too general. If active landscapes involve just the maintenance of biomass, it could be defined as “disturbance” in the context of “intermediate hypothesis”. But perhaps it might be more than just biomass.
2. Did this hypothesis explain the species abundance in both the native and the invaded range? Please check the third paragraph in the introduction with the third paragraph in the page four. From the manuscript I understand that it explains the abundance in both situations.
3. In the second paragraph of the introduction: I don’t find the aim of the last line. Moreover, the author could improve the first paragraph after the subtitle The Human Release Hypothesis, because I also don’t understand the logic among that paragraph. It seems to be two important sentences (important), but I don’t find any cohesion between them. It is also quite hard to understand what the paragraph aims for.
4. Future context. 1; how this hypothesis works for different life forms (competitor; stress-tolerator; ruderal plant strategy). I find really interesting thinking of this. Maybe the context of Fig. 1 of Diez and Edwards (2006) is a good scene. 2; the manuscript focus on Europe as the principal and the only example, but maybe Asia is another possible example. On one hand it provides a great amount of invasive species and on the other hand it has a higher proportion of dense settlements. Species like Cotoneaster, Ligustrum and Pyracantha that are native from Asia are the most invasive species in the earth and cover at least in Argentine, an significant portion of landscape.

I have read this submission. I believe that I have an appropriate level of expertise to confirm that it is of an acceptable scientific standard.

# Response

**Published:** 2014-12-09

**Authors:** Heike Zimmermann

**Affiliations:** Leuphana University, Germany

Thank you for your thorough and helpful review. We have incorporated all the minor comments concerning typos and wording. For our response to the mayor comments please see below: 

1. We included a more detailed explanation of landscape maintenance. The key difference between "disturbance" and "maintenance" is the time scale. Disturbance can be a single event but maintenance is defined as "work that is done to KEEP something in good conditions".
  See Introduction: "We define intermediate levels of human activity as activity patterns defined by sporadic disturbance events that are followed by long periods lacking active management, such as fallowing or abandonment. In contrast, regions with high levels of human activity frequently experience active management, such as weeding, hedge trimming or mowing of field margins." 
  See section The Human Release Hypothesis in the context of other invasion hypotheses: "Thus, we hypothesize that the abundance of invasive species should be highest in between these two extremes – namely in extensively used landscapes characterized by frequent fallowing, low levels of weed control, high heterogeneity, and many disturbed edges of small farmland patches ^32^. Such landscapes are where “human release” should contribute to optimal conditions for invasive species to establish large populations."
2. Yes, it does explain the abundance in both ranges. We clarified this now further in the Introduction: "Finally, we propose that the Human Release Hypothesis can also explain why some species that are highly abundant in their invasive range have relatively low abundance in their native range."
3. We re-wrote this paragraph and hope it is now comprehensible:
  "To date, extensive data on the abundance of invasive alien species is widely lacking. Existing approaches to predict invasion patterns in response to anthropogenic global change have focused primarily on the development of novel ecosystems ^11^ and alien species richness ^12^. Based on this, it is now widely acknowledged that systems containing high numbers of alien species tend to be those created and sustained by humans."
4. Future context. We now included a paragraph on how our hypothesis applies to different life strategies, and we encourage to investigate how comparisons between species from Asia and their invasive range could fit to our hypothesis. 
  See section The Human Release Hypothesis in the context of other invasion hypotheses: "Disturbance events also provide windows of opportunity for invasive species ^26^ and are often the result of human activity. Many invasive plant species are adapted to exploit temporarily favourable conditions through their short life cycles, rapid growth, high reproductive allocation, persistent soil seed banks and rapid germination (the Ideal Weed Hypothesis) ^27^. All these traits are also of advantage in systems where frequent weeding or mowing is practiced. Therefore, species pursuing this competitive ruderal strategy could profit twofold from Human Release."
  See last section: "We generated our hypothesis based on findings in Europe, however many invasive plant species on the American continent originate from Asia ^42,43^, thus it would be interesting to test our hypothesis based on land use patterns from these regions. "

# Peer review: Referee response for version 1

**DOI:** 10.5256/f1000research.4008.r4769

**Published:** 2014-05-23

**Authors:** Christoph Kueffer

**Affiliations:** 1 Institute of Integrative Biology, Swiss Federal Institute of Technology, Zurich, Switzerland

This is an interesting concept paper. It touches upon two timely research problems in invasion science: (i) how to better incorporate the role of humans into invasion theory, and (ii) how to improve the predictability of the abundance of invasive alien species (instead of only occurrence).

The key idea of the proposed ‘human release hypothesis’ seems that land management intensity – especially of abandoned or extensively used habitat – can explain differences in the abundance of a species between its native and alien ranges because the abundance of the species might be reduced in the native range through more intensive management of such land and associated cutting of the species. This is an interesting idea because it states that the presence of humans and their effects on landscapes can reduce invasion spread in opposition to traditional thinking that sees human land use mainly as a driver of invasions. The authors propose for instance that differences in land management coverage and intensity might explain why Europe is less invaded than regions with a higher proportion of wildlands such as North America or Australia.

The argumentation of the article fits also well with recent thinking in biodiversity conservation that emphasizes that permanent conservation intervention will in the future increasingly be necessary to maintain threatened biodiversity and ecosystem services in an era of global change; and that this will require building on synergies with other land use practices (such as weeding through agricultural practices in buffer zones around protected areas).

I have read this submission. I believe that I have an appropriate level of expertise to confirm that it is of an acceptable scientific standard.
